# Retraction: A phase II, single-arm study of apatinib and oral etoposide in heavily pre-treated metastatic breast cancer

**DOI:** 10.3389/fonc.2026.1798367

**Published:** 2026-02-10

**Authors:** Frontiers Editorial Office

Following publication, significant concerns were raised regarding the integrity of the patient data and reporting within the manuscript. An investigation was conducted in accordance with Frontiers’ policies, and it was determined that the conclusions and assertions were not sufficiently supported by the findings from the material provided; therefore, the article has been retracted.

This retraction was approved by the Chief Editors of Frontiers in Oncology and the Chief Executive Editor of Frontiers. The authors agree to this retraction.

